# A validation of an entropy-based artificial intelligence for ultrasound data in breast tumors

**DOI:** 10.1186/s12911-023-02404-z

**Published:** 2024-01-02

**Authors:** Zhibin Huang, Keen Yang, Hongtian Tian, Huaiyu Wu, Shuzhen Tang, Chen Cui, Siyuan Shi, Yitao Jiang, Jing Chen, Jinfeng Xu, Fajin Dong

**Affiliations:** 1 The Second Clinical Medical College, Jinan University, 518020 Shenzhen, China; 2 Research and development department, Illuminate, LLC, 518000 Shenzhen, Guangdong China; 3 Shenzhen People’s Hospital, 518020 Shenzhen, China

## Abstract

**Background:**

The application of artificial intelligence (AI) in the ultrasound (US) diagnosis of breast cancer (BCa) is increasingly prevalent. However, the impact of US-probe frequencies on the diagnostic efficacy of AI models has not been clearly established.

**Objectives:**

To explore the impact of using US-video of variable frequencies on the diagnostic efficacy of AI in breast US screening.

**Methods:**

This study utilized different frequency US-probes (L14: frequency range: 3.0-14.0 MHz, central frequency 9 MHz, L9: frequency range: 2.5-9.0 MHz, central frequency 6.5 MHz and L13: frequency range: 3.6-13.5 MHz, central frequency 8 MHz, L7: frequency range: 3-7 MHz, central frequency 4.0 MHz, linear arrays) to collect breast-video and applied an entropy-based deep learning approach for evaluation. We analyzed the average two-dimensional image entropy (2-DIE) of these videos and the performance of AI models in processing videos from these different frequencies to assess how probe frequency affects AI diagnostic performance.

**Results:**

The study found that in testing set 1, L9 was higher than L14 in average 2-DIE; in testing set 2, L13 was higher in average 2-DIE than L7. The diagnostic efficacy of US-data, utilized in AI model analysis, varied across different frequencies (AUC: L9 > L14: 0.849 vs. 0.784; L13 > L7: 0.920 vs. 0.887).

**Conclusion:**

This study indicate that US-data acquired using probes with varying frequencies exhibit diverse average 2-DIE values, and datasets characterized by higher average 2-DIE demonstrate enhanced diagnostic outcomes in AI-driven BCa diagnosis. Unlike other studies, our research emphasizes the importance of US-probe frequency selection on AI model diagnostic performance, rather than focusing solely on the AI algorithms themselves. These insights offer a new perspective for early BCa screening and diagnosis and are of significant for future choices of US equipment and optimization of AI algorithms.

**Supplementary Information:**

The online version contains supplementary material available at 10.1186/s12911-023-02404-z.

## Key points

1. The study explored the impact of ultrasound images with different frequencies on the diagnostic efficacy of artificial intelligence.

2. Ultrasound images obtained with different frequency probes exhibited variable levels of average two-dimensional image entropy, influencing the diagnostic performance of artificial intelligence models in nuanced ways.

3. Datasets with higher average two-dimensional image entropy were associated with superior artificial intelligence breast diagnostic efficacy.

## Abstract (fr)

The research on artificial intelligence-assisted breast diagnosis often relies on static images or dynamic videos obtained from ultrasound probes with different frequencies. However, the effect of frequency-induced image variations on the diagnostic performance of artificial intelligence models remains unclear. In this study, we aimed to explore the impact of using ultrasound images with variable frequencies on AI’s diagnostic efficacy in breast ultrasound screening. Our approach involved employing a video and entropy-based feature breast network to compare the diagnostic efficiency and average two-dimensional image entropy of the L14 (frequency range: 3.0-14.0 MHz, central frequency 9 MHz), L9 (frequency range: 2.5-9.0 MHz, central frequency 6.5 MHz) linear array probe and L13 (frequency range: 3.6-13.5 MHz, central frequency 8 MHz), and L7 (frequency range: 3-7 MHz, central frequency 4.0 MHz) linear array probes. The results revealed that the diagnostic efficiency of AI models differed based on the frequency of the ultrasound probe. It is noteworthy that ultrasound images acquired with different frequency probes exhibit different average two-dimensional image entropy, while higher average two-dimensional image entropy positively affect the diagnostic performance of the AI model. We concluded that a dataset with higher average two-dimensional image entropy is associated with superior diagnostic efficacy for AI-based breast diagnosis. These findings contribute to a better understanding of how ultrasound image variations impact AI-assisted breast diagnosis, potentially leading to improved breast cancer screening outcomes.

## Introduction

Breast cancer (BCa) is the most prevalent cancer and the leading cause of cancer mortality in females worldwide [1, 2]. Early identification and intervention of BCa lead to significant improvements in 5-year relative survival rates [3, 4]. Ultrasound(US) is the imaging method of choice for the evaluation of breast disease because it is technically simple, cost-effective, and safe [5]. Also, the US is seen as a primary measure of BCa detection and mortality reduction [3]. However, US-image-based diagnosis of the breast greatly relies on the experiences of the sonographers [6], so it is significant to further explore the information carried by US images to enhance the detection rate and diagnostic accuracy of BCa in its early stages.

In recent years, artificial intelligence (AI) has brought opportunities for the advancement of medical imaging [7–10]. The algorithm is enabled to extract a large amount of information from medical images that cannot be observed by the naked eye for diagnosis and improve the computer detection rate of nodules [11–13]. US-based AI studies rely on the sonographers-selected images during the scanning process or partially on responsibility frames selected from video [14–21]. Therefore, the US image selection is particularly crucial in BCa AI diagnosis.

In information theory [22], entropy is the average amount of information contained in each received “message”. Image entropy (IE) is a statistical form of image features, reflecting the average amount of information in the image, which can reflect the distribution complexity of each pixel point of the image [23]. Most previous studies were image-based that required high-frequency probe acquisition as a dataset [24–26]. The principle of choosing a US probe is to ensure sufficient detection depth while maximizing the frequency to ensure the resolution of the US image [25]. Although the high-frequency probe images may aid the sonographer in making a diagnosis. Whether they are favorable for the training and diagnosis of AI models is not known yet. Earlier works [21] demonstrated that the richer the average information content of an image, the better its tumor classification. Thus, based on the principle that US low frequency corresponds to high penetration [27], US data obtained at different frequencies may carry different levels of information, thereby impacting the diagnosis of the AI model.

Therefore, this study introduces the feature entropy of breast US to calculate the magnitude of the average two-dimensional image entropy(2-DIE) at different frequencies. Further, to investigate whether the US images obtained at lower frequencies have higher average 2-DIE and are more beneficial to improve AI diagnosis.

## Materials and methods

### Participants

This retrospective research collected US videos examined at Shenzhen People’s Hospital from June 2021 to December 2021. As a retrospective study, informed consent was waived by the Medical Ethics Committee of Shenzhen People’s Hospital. All patient information was handled with strict confidentiality in compliance with ethical guidelines. The benignity and malignancy of the nodules obtained by the US were confirmed by pathology.

#### Inclusion criteria

(a) Simultaneous acquisition of US video images of tumors in the same patient with two different frequency probes. (b) US-detected nodules must be classified as 0, 2, 3, 4a, 4b, 4c, or 5 following the BI-RADS. (c) No biopsy or surgical treatment of the breast nodules is to be evaluated before the US scan. (d) Patients were biopsied or surgically treated within 1 week of US data acquisition, while pathological results were obtainable.

#### Exclusion criteria

(a) BI-RADS 6 in the US, DR (Digital mammography), or MRI (magnetic resonance imaging). (b) BI-RADS 1 in the US. (c) History of breast surgery. (d) Single-frequency US video data. (e) Missing pathological results. (f) Poor image quality.

In this study, a total of 668 breast tumors (260 malignancy and 408 benign) of US videos from 167 female patients were included and divided into 2 testing sets: (1) In testing set 1, breast US videos were obtained from Resona I9 (Mindray, China) with L14 (frequency range: 3.0-14.0 MHz, central frequency 9 MHz), L9 (frequency range: 2.5-9.0 MHz, central frequency 6.5 MHz) linear array probe. (2) Testing set 2, the data were obtained from DC-65 (Mindray, China) with L13 (frequency range: 3.6-13.5 MHz, central frequency 8 MHz), and L7 (frequency range: 3.0-7.0 MHz, central frequency 4.0 MHz) linear array probe, which was aimed to further evaluate our theory and discoveries.

### Ultrasound examination and video acquisition

In this study, all US videos were acquired by the same radiologist with more than 10 years of experience. The researchers utilized 3 markers to localize the location of the mass during the collection process. Specifically, the largest section of the target tumor was first located using one of the frequency probes and marked on the body surface. Then a complete sweep was made horizontally along the largest section of the tumor to find two more markers ≥ 2 cm from the tumor margin, respectively. Finally, the whole tumor is swept along the marked direction, and the operation is repeated, keeping the direction and position of the probe consistent each time, until the US video acquisition of the four different frequencies is completed.

### Processing of US-video and use of AI model

**Fig. 1:**
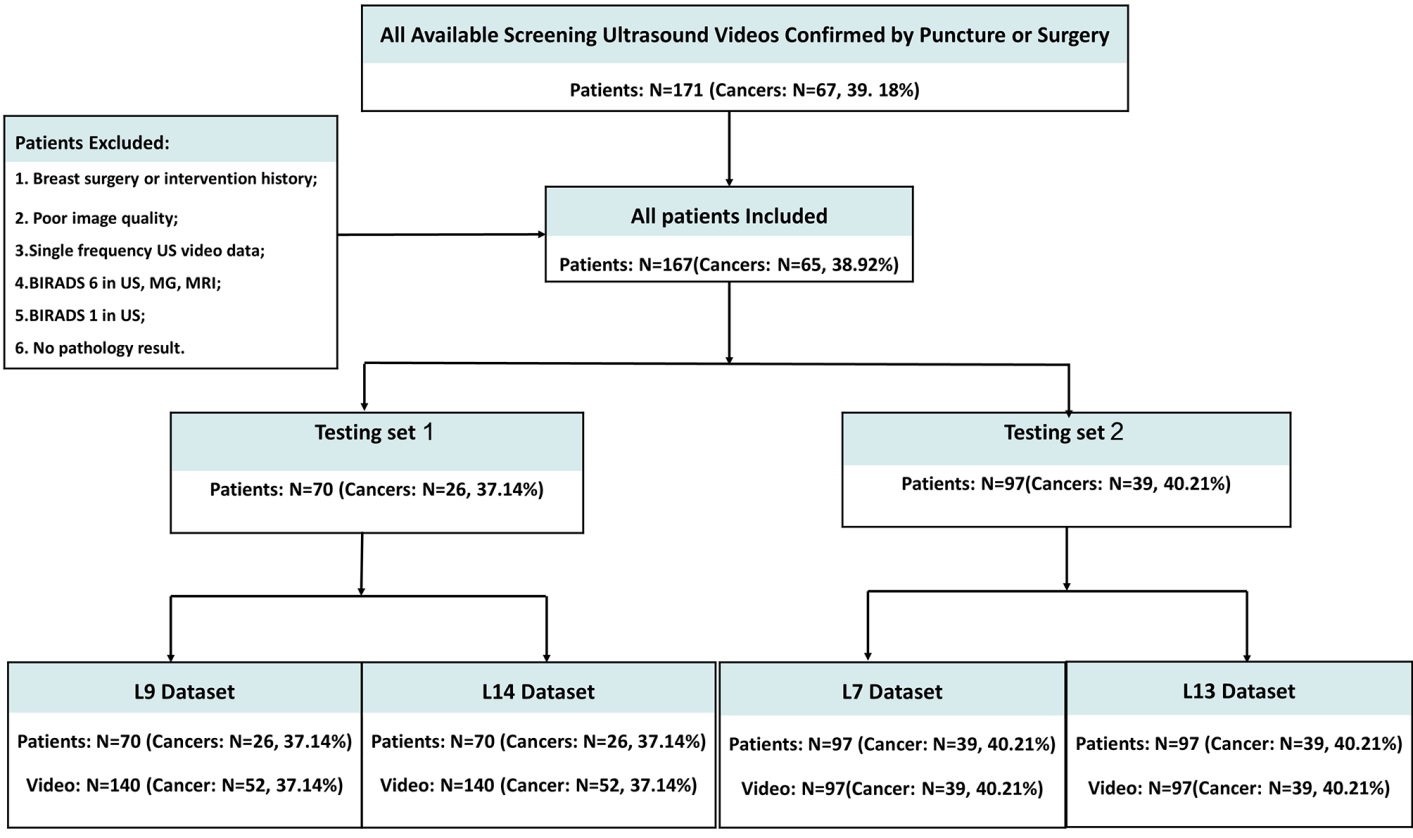
The flow charts of this Study

First, we conducted this study based on the constructed feature entropy breast network (FEBrNet), which inherits the pre-trained backbone of the fully connected layer and the weight-optimal model [21]. We use the AI model to select responsibility frames to reduce subjective dependence. Our method of selecting pivotal frames draws inspiration from established applications of entropy in information theory, such as in decision trees. Specifically, the Iterative Dichotomiser 3 (ID3) decision tree algorithm utilizes entropy to ascertain the most suitable parent node and its division. In our methodology, we aim to minimize the discrepancy between the FScore of the video and that of the chosen frame collection, where a smaller disparity indicates that the information content of the chosen frames closely mirrors that of the entire video. By incrementally adding frames to this collection, starting from one and increasing to n, and at each increment selecting the frame that least differs, we gradually form an optimally representative set of frames, each contributing unique features. Subsequently, for the final collection of these optimal frames, our study computes the two-dimensional image entropy for each frame using the FEBrNet model. We then determine the video’s image entropy by calculating the average two-dimensional image entropy (2-DIE) of all the chosen frames. Finally, pathological results were used as the gold standard to compare the ability of using image entropy of different frequencies in the differential diagnosis of benign and malignant breast tumors. The processing and validation of the data are based on the pre-trained entropy-based model (FEBrNet). For specific information about the model refer to this literature [21] and supplementary materials. We investigate and verify the effect of entropy on the diagnostic performance of AI models from the perspective of IE. US images obtained from US probes of different frequencies are various. The researchers compared the variations by collecting US data from the same patient at different frequencies simultaneously. This is used to research the difference in diagnostic efficacy of US images obtained at different frequencies for AI models. The flow chart is shown in Fig. 1.

### Statistical analysis

Statistical analysis was performed using R 3.6.3 (Copyright (C) 2020 The R Foundation for Statistical Computing). The significance level was set at *P* < 0.05. A normality test was performed for each variable. T-test is used for the normally distributed numerical variables, the rank sum test is used for the non-normally distributed numerical variables, and the Chi-square test is used for the disordered classification variables. The paired sample t-test was used to compare the differences within the group. The specificity, sensitivity, accuracy, receiver operating characteristic curve (ROC), and area under the curve (AUC) were used to evaluate models.

## Results

### Participant characteristics

According to the inclusion and exclusion criteria, A total of 668 tumor videos from 167 patients were included in this study, including 260 videos of cancerous masses and 408 videos of benign tumors. There are 280 videos in the testing set 1 and 388 videos in testing set 2. Table 1 show the baseline distribution characteristics of the collected patients, respectively.

**Table 1:** The distribution of baseline characteristics based on testing sets

**Testing set 1**				
**Variables**	**Total** **(n = 70)**	**Benign** **(n = 44)**	**Malignant** **(n = 26)**	***p***
Age, Mean ± SD	43.29 ± 12.9	38.89 ± 11.42	50.73 ± 11.99	< 0.01
Height, Mean ± SD	159.17 ± 3.86	159.57 ± 3.86	158.5 ± 3.85	0.27
Weight, Mean ± SD	57.49 ± 6.97	56.27 ± 6.42	59.54 ± 7.5	0.07
BI-RADS, n (%)				< 0.001
2	14 (20)	14 (32)	0 (0)	
3	14 (20)	14 (32)	0 (0)	
4 A	11 (16)	9 (20)	2 (8)	
4B	14 (20)	7 (16)	7 (27)	
4 C	8 (11)	0 (0)	8 (31)	
5	9 (13)	0 (0)	9 (35)	
Max. size, Median (Q1, Q3)	8 (6, 14)	7 (5.75, 11)	12.5 (8, 15)	0.001
**Testing set 2**				
**Variables**	**Total** **(n = 97)**	**Benign** **(n = 58)**	**Malignant** **(n = 39)**	***p***
Age, Median (Q1, Q3)	40 (31, 50)	36 (28.5, 41)	49 (43, 58.5)	< 0.01
Height, Median (Q1, Q3)	158 (155, 162)	159 (156, 2.75)	158 (155, 162)	0.87
Weight, Median (Q1, Q3)	57 (52, 62)	55.5 (51, 60)	60 (56.5, 64.5)	< 0.01
BI-RADS, n (%)				< 0.01
2	20 (21)	20 (34)	0 (0)	
3	19 (20)	19 (33)	0 (0)	
4 A	14 (14)	13 (22)	1 (3)	
4B	15 (15)	6 (10)	9 (23)	
4 C	15 (15)	0 (0)	15 (38)	
5	14 (14)	0 (0)	14 (36)	
Max. size, Median (Q1, Q3)	13 (9, 23)	11.5 (7, 18.75)	17 (12.5, 24)	< 0.01

Note: BI-RADS: Breast Imaging-Reporting and Data System

### Distribution of 2-DIE in various frequencies

**Fig. 2:**
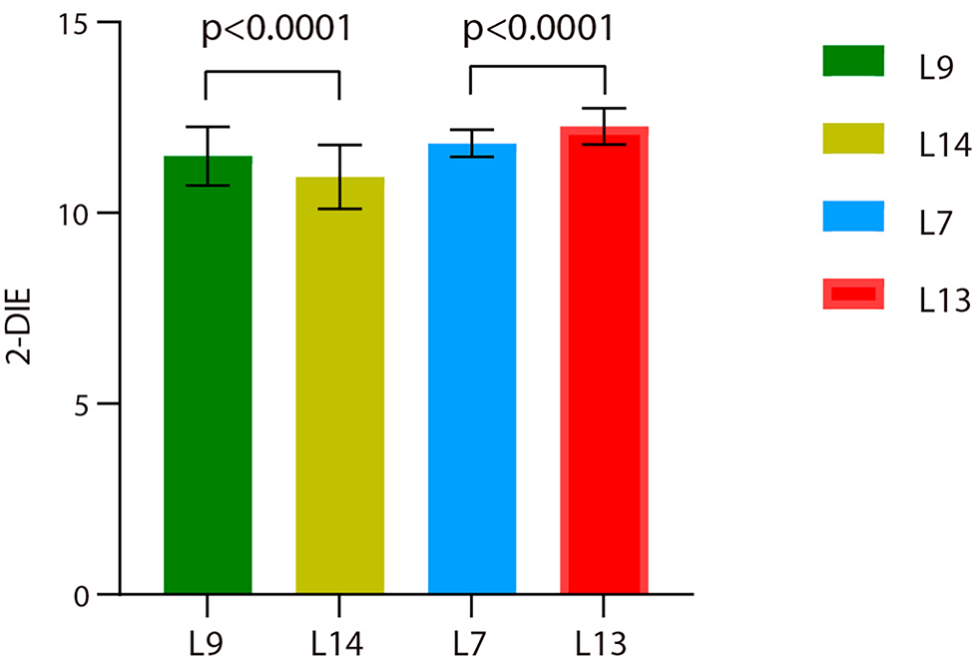
The results of the average 2-DIE of the two testing sets. Note: 2-DIE: two-dimensional image entropy (unless otherwise stated, the above measurements are average values); L9: L9 linear array probe, frequency range: 2.5-9.0 MHz, central frequency 6.5 MHz; L14: L14 linear array probe, frequency range: 3.0-14.0 MHz, central frequency 9 MHz; L7: L7 linear array probe, frequency range: 3-7 MHz, central frequency 4.0 MHz; L13: L13 linear array probe, frequency range: 3.6-13.5 MHz, central frequency 8 MHz; P: L9 vs. L14, L7 vs. L13

The values of the average 2-DIE obtained at different frequencies are variable. For testing set 1, the 2-DIE of the L9 linear probe was higher than that of L14 (Mean ± SD,11.49 ± 0.769 vs. 10.94 ± 0.835); For the testing set 2, the 2-DIE of the L13 linear probe was higher than that of L7 (Mean ± SD,11.82 ± 0.356 vs. 12.27 ± 0.476). This result is summarized in Fig. 2.

### Diagnosis performance of AI models

**Fig. 3:**
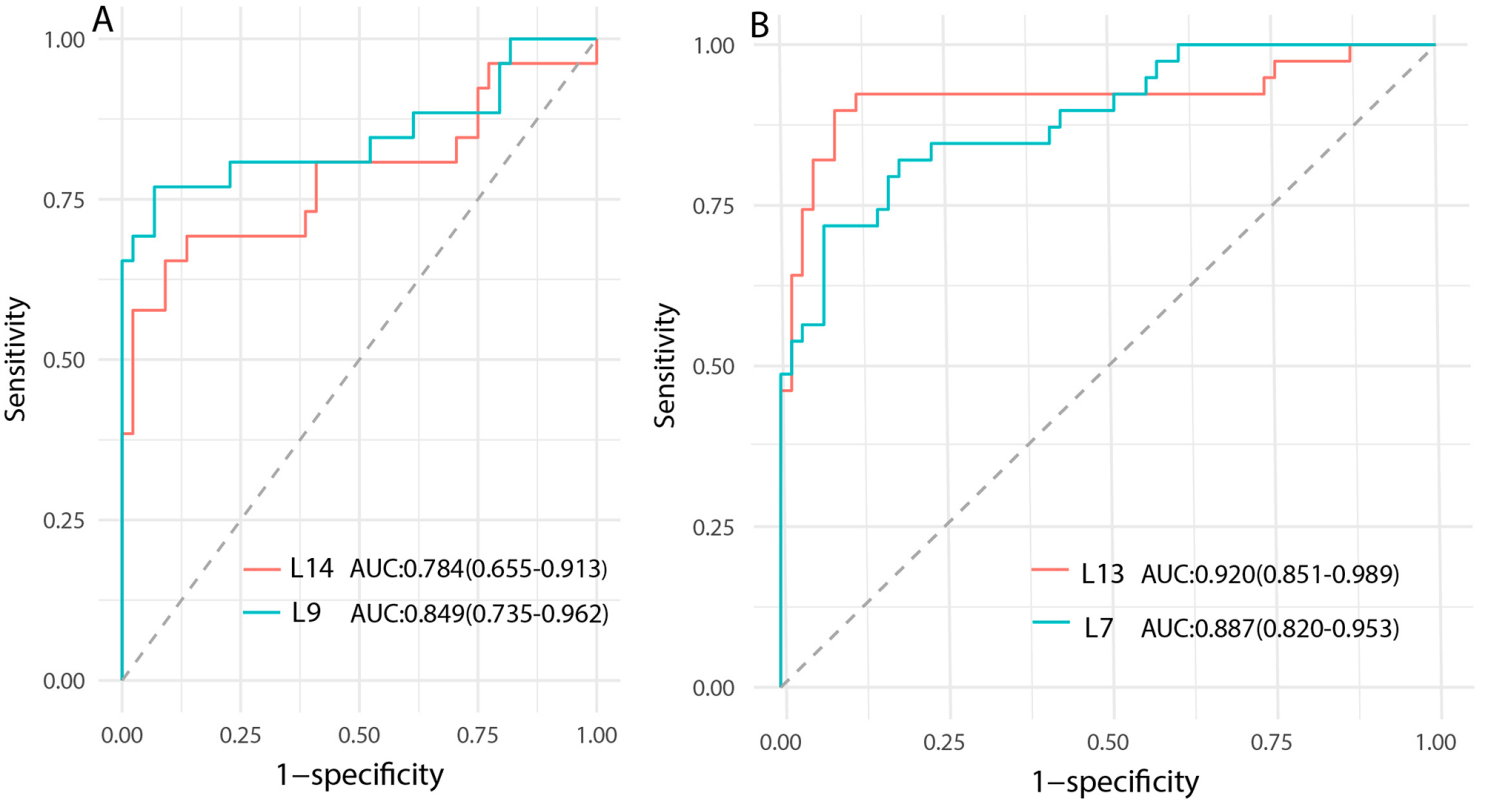
Comparison of diagnostic performance of the testing sets. Note: AUC: area under the curve; 95% CI: 95% confidence interval; L9: L9 linear array probe; L14: L14 linear array probe; L7: L7 linear array probe; L13: L13 linear array probe. (**A**): Testing 1; (**B**): Testing 2

The diagnostic efficacy of US data for AI models varies at distinct frequencies. For the testing set 1, L9 attained the best AUC (0.849), with a sensitivity of 76.9%, specificity of 93.2%, and accuracy of 87.1%. For the testing set 2, L13 reached the best AUC (0.920), sensitivity 89.7%, specificity 93.8%, as well as accuracy 91.0%. The detailed results are shown in Table 2; Fig. 3.

**Table 2:** Comparison of the efficacy of AI models

Model	AUC (95%CI)	Sensitivity (%)	Specificity (%)	Accuracy (%)	*P* value
L9	0.849 (0.735–0.962)	76.9	93.2	87.1	0.4185^#^
L14	0.784 (0.655–0.913)	65.4	90.9	81.4	
L7	0.887 (0.820–0.953)	71.8	93.4	85.0	0.0383^*^
L13	0.920 (0.851–0.989)	89.7	91.8	91.0	

Note: AUC: area under the curve; 95% CI: 95% confidence interval; L9: L9 linear array probe, frequency range: 2.5-9.0 MHz, central frequency 6.5 MHz; L14: L14 linear array probe, frequency range: 3.0-14.0 MHz, central frequency 9 MHz; L7: L7 linear array probe, frequency range: 3-7 MHz, central frequency 4.0 MHz; L13: L13 linear array probe, frequency range: 3.6-13.5 MHz, central frequency 8 MHz; *P* value #: L9 vs. L14; *: L7 vs. L13

## Discussion

In this study, we used a video and entropy-based deep learning model [21] to compare the diagnosis performance of breast US. The assessment effect of variable frequencies on the AI model diagnosis validity was based on two retrospective data sets (Mindray L7/L13 and L9/L14). In testing set 1, compared to L14 (frequency range: 3.0-14.0 MHz, central frequency 9 MHz), the L9-had better diagnosis performance and 2-DIE. However, in testing set 2, compared to L7 (frequency range: 3-7 MHz, central frequency 4.0 MHz), the L13-had better diagnosis performance and 2-DIE. This observation suggests that US-data derived from probes operating at varying frequencies can significantly impact the diagnostic effectiveness of AI models. Another finding is that higher 2-DIE is accompanied by increased diagnostic efficacy.

In recent years, many AI-based studies have investigated the benign and malignant categorization of US breast nodules [18, 28–37]. The accuracy of their models fluctuates from 80 to 95%. While the literature recommends a frequency range of 5–17 [38] for breast US screening, it does not specify which one to use. Also, there is no literature examining the distinction in the diagnostic utility of AI for images acquired by various probes. The probe frequencies used in the studies in the literature mentioned above ranged from 1 to 42 MHz. Therefore, one reason for the variation in accuracy between these surveys may be the variance in the frequency of the probes utilized. So, we did this experience and discovered that the L9 (frequency range: 2.5-9.0 MHz, central frequency 6.5 MHz) had better diagnosis performance and higher 2-DIE. This may be contradicted by our clinical experience but offers another probability. While high-frequency US probes typically require more sophisticated technology and may be costlier, their application might not always correspond to improved diagnostic performance in AI models. Furthermore, primary hospitals may be unable to afford the purchase and maintenance of high frequency probe. The results of this experiment may now solve this challenge – using AI to aid diagnosis and compensate for the low accuracy of clinicians when using low frequency probe. On the other hand, excessively low-frequency probes do not enhance the diagnostic performance of AI models. In the testing set 2, we found that the L13 (frequency range: 3.6-13.5 MHz, central frequency 8 MHz) had better diagnosis performance. This is inconsistent with the results of our testing set 1. Possible reasons for this result include (a. The penetration is excessive, resulting in images that contain more confounding information unrelated to the lesion. b. The high frequency probe provides excellent spatial and soft-tissue resolution, greatly improving the differentiation of lesion saliency. However, the results for the 2-DIE of the L13 are higher than those of the low-frequency ones, which remains consistent with our previous findings.

Also, previous studies [21] by our team demonstrated that the richer the 2-DIE contained in US images, the more favorable the prediction of breast tumor benignity-malignancy. That is, a high 2-DIE in US images corresponds to rich image information. Meanwhile, US features varied depending on the pathological heterogeneity of the breast tumor [39]. The richer the information contained in the US image, the more comprehensive the information it may contain about the tumor characteristics. Moon et al. [34] also indicated that images with more information would help improve the diagnostic efficacy of the model. The results of this experiment are consistent with previous studies – both in the two sets, the higher the 2-DIE, the better the diagnostic performance. Because of the higher penetration of the low frequency probe, visualization of deep posterior tissues is made easy. More information related to the nodules may be captured. This information may not be recognized by the naked eye but facilitates machine learning. Accompanying the development of AI and the concept of medical-industrial integration, the application of AI-assisted diagnosis may become more extensive. However, previous research has focused more on the innovation and refinement of algorithms and hardware, ignoring the differences in images of different frequencies. Therefore, it is necessary to investigate the diagnostic efficacy of different frequency datasets on AI models. Images acquired at more appropriate frequencies will help improve diagnostic performance and provide a reference for future US image acquisition for AI models.

There are some limitations in this study. First, the study was a retrospective single-center study with smaller sample size and uneven image quality. Second, Lack of comparison of diagnostic efficacy of different frequencies for AI models. Therefore, we will further investigate the effect of other frequencies on AI diagnostic efficacy in the next research plan. Finally, Variations in sensitivity and inter-machine variability of various US devices were not considered.

## Conclusion

This study indicate that US-data acquired using probes with varying frequencies exhibit diverse average 2-DIE values, and datasets characterized by higher average 2-DIE demonstrate enhanced diagnostic outcomes in AI-driven BCa diagnosis. Unlike other studies, our research emphasizes the importance of US-probe frequency selection on AI model diagnostic performance, rather than focusing solely on the AI algorithms themselves. These insights offer a new perspective for early BCa screening and diagnosis and are of significant guidance for future choices of US equipment and optimization of AI algorithms.

### Electronic supplementary material

Below is the link to the electronic supplementary material.

Supplementary Material 1

## Data Availability

The datasets used and analyzed during the current study are available from the corresponding author on reasonable request.

## Data Availability

The datasets used and analyzed during the current study are available from the corresponding author on reasonable request.

## Abbreviations

BCa: breast cancer
2-DIE: two-dimensional image entropy
US: ultrasound
AI: artificial intelligence
IE: Image entropy
DR: Digital mammography
MRI: magnetic resonance imaging
L9: L9 linear array probe (frequency range:2.5-9.0 MHz, central frequency 6.5 MHz)
L14: L14 linear array probe (frequency range:3.0-14.0 MHz, central frequency 9 MHz)
L7: L7 linear array probe (frequency range:3-7 MHz, central frequency 4.0 MHz)
L13: L13 linear array probe (frequency range:3.6-13.5 MHz, central frequency 8 MHz)
BI-RADS: Breast Imaging-Reporting and Data System
ROC: receiver operating characteristic curve
CI: confidence interval
AUC: area under the curve
IQR: interquartile range
