# Supplementary material for: A validation of an entropy-based artificial intelligence for ultrasound data in breast tumors
Source: BMC Med Inform Decis Mak. 2024 Jan 2;24:1. doi: 10.1186/s12911-023-02404-z (PMC10759705; doi:10.1186/s12911-023-02404-z)
Supplement: Supplementary file 1 — Supplementary Material 1 [file 12911_2023_2404_MOESM1_ESM.docx]

**Entropy-based deep learning models**

First, we conducted this study based on the constructed feature entropy breast network (FEBrNet), which inherits the pre-trained backbone of the fully connected layer and the weight-optimal model. We use the AI model to select responsibility frames to reduce subjective dependence. Our method of selecting pivotal frames draws inspiration from established applications of entropy in information theory, such as in decision trees. Specifically, the Iterative Dichotomiser 3 (ID3) decision tree algorithm utilizes entropy to ascertain the most suitable parent node and its division. In our methodology, we aim to minimize the discrepancy between the FScore of the video and that of the chosen frame collection, where a smaller disparity indicates that the information content of the chosen frames closely mirrors that of the entire video. By incrementally adding frames to this collection, starting from one and increasing to n, and at each increment selecting the frame that least differs, we gradually form an optimally representative set of frames, each contributing unique features. Subsequently, for the final collection of these optimal frames, our study computes the two-dimensional image entropy for each frame using the FEBrNet model. We then determine the video's image entropy by calculating the average two-dimensional image entropy (2-DIE) of all the chosen frames. Finally, pathological results were used as the gold standard to compare the ability of using image entropy of different frequencies in the differential diagnosis of benign and malignant breast tumors.

S-Figure 1 depicted a basic example of picking the top two responsible frames from a video with three frames and three feature dimensions. In step A, we create the video feature entropy matrix by applying MaxPooling across the three frame feature entropy matrices, resulting in an FScorevideo of 27. In step B, with the number of frames in the chosen frame collection set to one, we have three possible choices with FScores of 17, 22, and 13. Selecting frame2 is optimal here, as it yields the lowest difference between FScorevideo and Fscoreframe2. Step C increases the number of frames in the responsible frame collection to two and the first responsible frame(frame2) has already been chosen. FScore [frame2, frame1] is 22 and FScore [frame2, frame3] is 27, thus frame3 should be selected as the second responsible frame. Despite the fact that FScoreframe1 is larger, we will not choose frame1 as the second responsible frame for the benefit of adding frame1 to the responsible frame collection are few. Frame1 provides almost the same features as the already selected frame2, implying that they may also look similar.


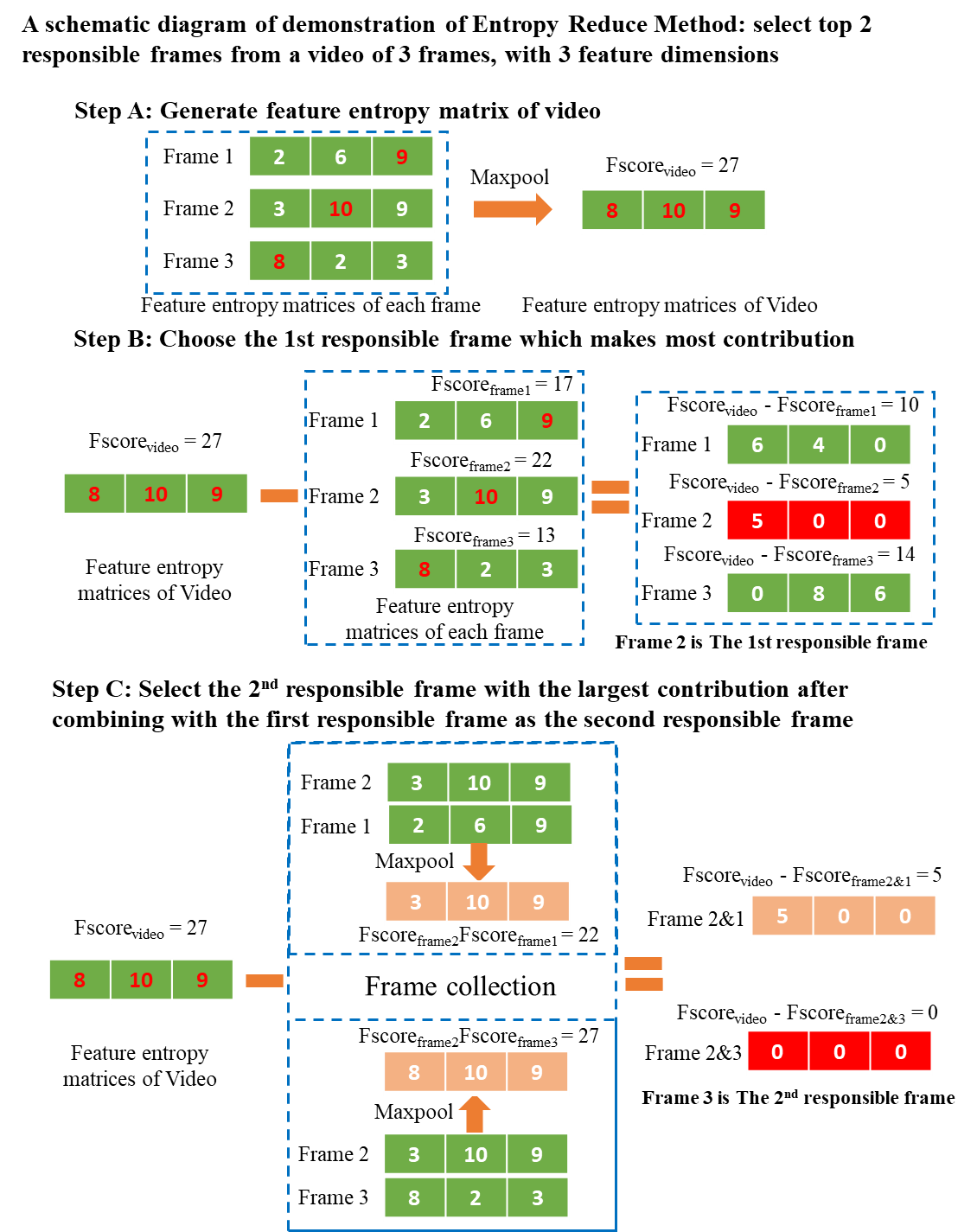


S-Figure 1 A demonstration of the Entropy Reduce technique applied for selecting key frames

Step A: MaxPool the three frame feature entropy matrices and yield the video feature entropy matrix. Step B: choose frame2 as the first responsible frame for it minimizing the difference between FScorevideo and FScoreframei. Step C: add another frame to the responsible frame collection, whereas step B already chose one. Since the FScore difference between the responsible frame collections of frame2 and frame3 is the smallest, frame3 is picked as the second responsible frame.

For more specific information and calculation formulas about the model, see published articles. Specific formulas have been published in IScience articles (DOI： 10.1016/j.isci.2022.105692).
